# Compend of Dental Pathology and Therapeutics

**Published:** 1897-05

**Authors:** 


"Compend of Dental Pathology and Therapeutics." By Henry H. Burchard, M. D., D. D. S., Special Lecturer upon Dental Pathology and Therapeutics in Philadelphia Dental College. Publisher: The S. S. White Dental Mfg. Co., Phila.

The object of the author of this compend was not to present a volume of questions and answers on dental pathology and therapeutics which will aid the student in memorizing answers for examinations. but rather to have each of them represent a guiding principle in such subjects; in other words "a student's note-book, as it is interlined with blank pages for additions and details to be derived from, or in conformity with didactic teachings. That the author of this small work has succeeded in accomplishing his object an examination of it will clearly prove. Such subjects as Hyperæmia, Inflammation, Bacteria, Fever, Septicemia and Pyemia, Embolus and Thrombus, Necrosis, Regeneration, Dental Histology, Anatomy and Embryology, Malformations of Teeth, Primary Dentition, Eruption of Permanent Teeth, Diseases of Enamel, Green Stain, Diseases of Dentine and Pulp, Dental Caries, Therapeutics of Caries,

Pulp-capping, Extirpation of Pulp, Diseases of Pericementum, Calcic Inflammation and Calculi, Pyorrhœa Alveolaris, Erosion of Teeth, Dental Pharmacology and Materia Medica and Contents of Dental Medicine Cabinet are treated by questions and answers, rendering the work a convenient, valuable and comprehensive compend, which cannot fail to aid the dental student and also the dental practitioner.

